# Clinical application of the “sellar barrier’s concept” for predicting intraoperative CSF leak in endoscopic endonasal surgery for pituitary adenomas with a machine learning analysis

**DOI:** 10.3389/fsurg.2022.934721

**Published:** 2022-09-08

**Authors:** J. F. Villalonga, D. Solari, R. Cuocolo, V. De Lucia, L. Ugga, C. Gragnaniello, J. I. Pailler, A. Cervio, A. Campero, L. M. Cavallo, P. Cappabianca

**Affiliations:** ^1^ Division of Neurosurgery, Department of Neurosciences, Reproductive and Odontostomatological Sciences, Universita’ degli Studi di Napoli Federico II, Naples, Italy; ^2^ LINT, Facultad de Medicina, Universidad Nacional de Tucumán, Tucumán, Argentina; ^3^ Department of Advanced Biomedical Sciences, Universita’ degli Studi di Napoli Federico II, Naples, Italy; ^4^ Department of Neurological Surgery, Swedish Neuroscience Institute, Seattle, WA, United States; ^5^ Departamento de Neurocirugía, FLENI, Buenos Aires, Argentina

**Keywords:** sellar barrier, pituitary adenoma, CSF leak, machine learning, skull base surgery

## Abstract

**Background:**

Recently, it was defined that the *sellar barrier* entity could be identified as a predictor of cerebrospinal fluid (CSF) intraoperative leakage. The aim of this study is to validate the application of the sellar barrier concept for predicting intraoperative CSF leak in endoscopic endonasal surgery for pituitary adenomas with a machine learning approach.

**Methods:**

We conducted a prospective cohort study, from June 2019 to September 2020: data from 155 patients with pituitary subdiaphragmatic adenoma operated through endoscopic approach at the Division of Neurosurgery, Università degli Studi di Napoli “Federico II,” were included. Preoperative magnetic resonance images (MRI) and intraoperative findings were analyzed. After processing patient data, the experiment was conducted as a novelty detection problem, splitting outliers (i.e., patients with intraoperative fistula, *n* = 11/155) and inliers into separate datasets, the latter further separated into training (*n* = 115/144) and inlier test (*n* = 29/144) datasets. The machine learning analysis was performed using different novelty detection algorithms [isolation forest, local outlier factor, one-class support vector machine (oSVM)], whose performance was assessed separately and as an ensemble on the inlier and outlier test sets.

**Results:**

According to the type of sellar barrier, patients were classified into two groups, i.e., strong and weak barrier; a third category of mixed barrier was defined when a case was neither weak nor strong. Significant differences between the three datasets were found for Knosp classification score (*p* = 0.0015), MRI barrier: strong (*p* = 1.405 × 10^−6^), MRI barrier: weak (*p* = 4.487 × 10^−8^), intraoperative barrier: strong (*p* = 2.788 × 10^−7^), and intraoperative barrier: weak (*p* = 2.191 × 10^−10^). We recorded 11 cases of intraoperative leakage that occurred in the majority of patients presenting a *weak sellar barrier* (*p* = 4.487 × 10^−8^) at preoperative MRI. Accuracy, sensitivity, and specificity for outlier detection were 0.70, 0.64, and 0.72 for IF; 0.85, 0.45, and 1.00 for LOF; 0.83, 0.64, and 0.90 for oSVM; and 0.83, 0.55, and 0.93 for the ensemble, respectively.

**Conclusions:**

There is a true correlation between the type of *sellar barrier* at MRI and its *in vivo* features as observed during endoscopic endonasal surgery. The novelty detection models highlighted differences between patients who developed an intraoperative CSF leak and those who did not.

## Introduction

Endoscopic endonasal approach, representing the most suitable technique (1), is indicated for the removal of lesions upon the endocrinological status and eventual neurological defects.

Pituitary macroadenomas present a predominantly vertical growth pattern and, albeit being round and soft, displace and compress pituitary gland tissue: the latter gets to be a part along with arachnoid and sellar diaphragm of the interface between the tumor and the supradiaphragmatic area (2). According to its anatomical features, it has been possible to define three categories of the “so-called sellar barrier” as seen at the preoperative MRI i.e., weak, mixed, and strong.

Rarely, pituitary adenomas may present inner features, such as hard/rubbery consistency, which might make lesion removal *via* standard endoscopic corridor more troublesome, leading to an increased risk of CSF leak (3–5).

Although postoperative CSF fistula in transsphenoidal pituitary surgery appears to be notably low as compared to extended skull base surgery, peculiar care and efforts have been given to this issue (6, 7). However, there are only few reports concerning the possible risk factors that can be detected preoperatively to predict an intraoperative CSF leakage (8–11). The *sellar barrier concept* and its role in predicting the risk of intraoperative CSF leakage has been recently introduced (12, 13) and confirmed in a clinical multicentric study (14). Radiomics, consisting of conversion of images into mineable data and subsequent analysis for decision support, has been gaining attention (15) in association with data mining and machine learning (ML) algorithms, aiding in the interpretation of a large amount of information produced. ML is a branch of artificial intelligence that includes algorithms capable of modeling themselves and improving accuracy by analyzing datasets, without prior explicit programming (16), thus leading to the creation of predictive models (17–21).

The aim of this study is to validate the application of the sellar barrier concept for predicting intraoperative CSF leak in endoscopic endonasal surgery for pituitary adenomas with a quantitative approach. MRI features of the sellar barrier were defined, with machine learning, as a predictor of CSF leakage in a series of patients with intra-suprasellar pituitary adenoma undergoing endoscopic endonasal surgery.

## Materials and methods

Patients with intra-suprasellar subdiaphragmatic pituitary adenoma scheduled for tumor resection *via* endoscopic endonasal approach at the Division of Neurosurgery, Università degli Studi di Napoli “Federico II,” from June 2019 to September 2020 were included in the study. Those who had a history of treatment for pituitary adenoma (radiation or medical therapy) or significant artifacts on the images used for the analysis were excluded.

### Preoperative MRI

All patients underwent radiological preoperative assessment with a specific MRI protocol for the sellar region that included sagittal and coronal slices in T1-weighted volumetric sequences, with and without contrast; with axial and sagittal slices of the sealing region in T2-weighted, fluid-attenuated inversion recovery (FLAIR), and Echo Spin gradient sequences (1.5 and 3.0 T resonator).

**Figure 1:**
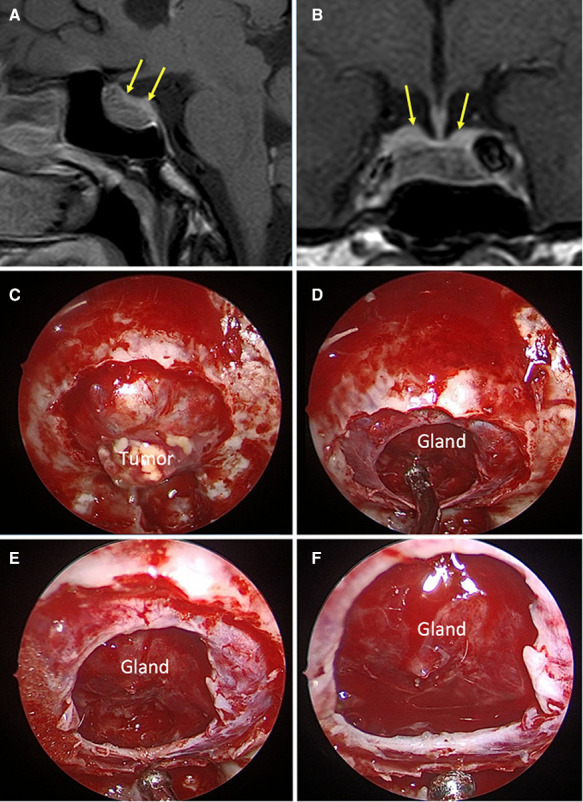
*Strong sellar barrier.* A 42-year-old male patient, with GH produce macroadenoma. (**A,B**) Preoperative MRI: the yellow arrows indicate the barrier that captures contrast with a thickness greater than 1 mm. (**C–F**) Intraoperative images: the barrier constituted by the gland can be seen.

**Figure 2:**
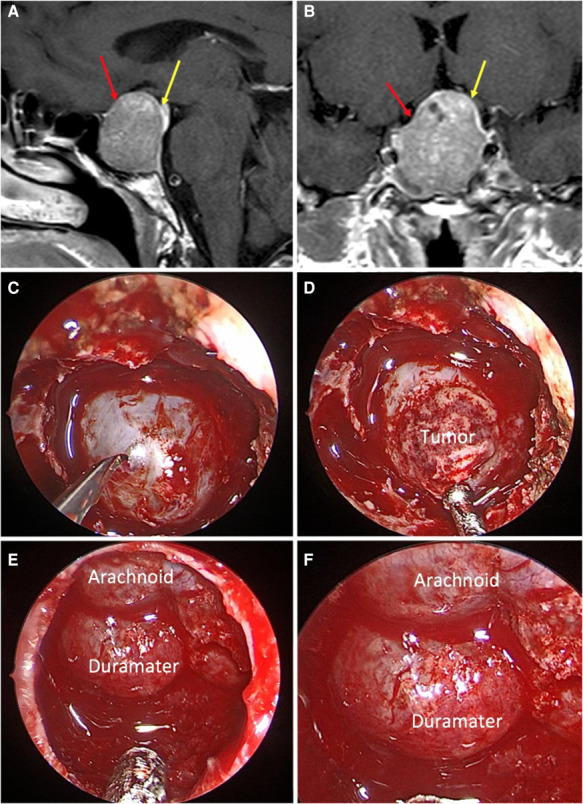
*Mixed sellar barrier.* A 59-year-old female patient, with a PRL produced macroadenoma. (**A,B**) Preoperative MRI: the yellow arrows indicate the barrier that captures contrast with a thickness greater than 1 mm and the red arrows indicate the barrier that captures contrast with a thickness less than 1 mm. (**C–F**) Intraoperative images: the barrier constituted by duramater and arachnoid can be seen.

**Figure 3:**
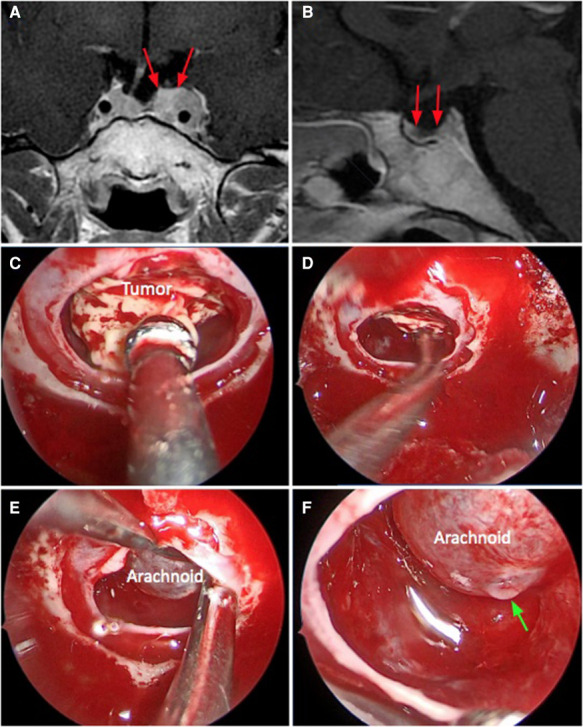
*Weak sellar barrier.* A 31-year-old female patient, with an ACTH produced macroadenoma. (**A,B**) Preoperative MRI: the red arrows indicate the barrier that captures contrast with a thickness less than 1 mm. (**C–F**) Intraoperative images: the barrier constituted only with arachnoid can be seen. The green arrow marks the CSF leak.

Considering the T1-weighted volumetric sequences, the evaluations of the *sellar barrier* were made with the Horos for Mac-OSX (Apple, California, USA). The measurements were made as explained in previous publications (2, 12–14). In each case, a neurosurgery resident classified the sellar barrier based on the MRI into three subtypes: strong barrier (greater than 1 mm), weak barrier (less than 1 mm), and mixed barrier (in the cases of coexistence of the two previous subtypes) (Figures 1–3, parts A,B).

#### Intraoperative management and findings

The surgeries were performed by the Senior authors of the Naples team (PC, LMC, DS) *via* an endoscopic endonasal standard approach (22–24). A Karl Storz / Co (Tuttlingen, Germany) endoscope with 0° lens was used as the sole visualizing tool; the four hands technique was adopted from the sphenoid phase. During each surgery, the Senior surgeon pointed out the sellar barrier type upon the intraoperative observation of gland and/or dura mater (strong barrier), only arachnoid tissue (weak barrier), and mixed components (mixed barrier). The eventual presence of intraoperative CSF leak was recorded according to the classification of Esposito et al. (7).

#### Feature engineering and preprocessing

Patient data were processed based on their nature using the pandas, numpy, and scikit-learn Python packages (25). Ordinal data (modified Knosp classification score) (26) were treated as a continuous variable to avoid loss of information. The presence of presurgical treatment was dichotomized. Then, categorical data were one-hot encoded using the pandas “get_dummy” function, converting *k* categories to *k* − 1 indicator variables. The final feature set comprised the following:

1.  Age
2.  Knosp classification score
3.  Gender
4.  Status: Growth hormone (GH) secreting
5.  Status: GH Prl secreting
6.  Status: Prl secreting
7.  Status: Nonfunctioning
8.  Size: Microadenoma
9.  MRI barrier: Strong
10.  MRI barrier: Weak
11.  Intraoperative barrier: Strong
12.  Intraoperative barrier: Weak
13.  Presurgical treatment

Given the distribution of classes within the datasets, the experiment was treated as a novelty detection problem. Therefore, outliers (i.e., patients with intraoperative fistula) and inliers were split into separate datasets. Then, the latter was further separated with an 80%/20% proportion into training and inlier test datasets. The only variable with missing values within all datasets was the modified Knosp classification score (25 missing values in the training set, 5 missing values in the inlier training set, and no missing values in the outlier test set). An imputer based on the mode of this parameter was fit on the training set (mode = 2) and used to transform all datasets to remove the missing values. Then, continuous variables were normalized using a min–max scaler (range = 0–1), also fit exclusively on the training data and used to transform all datasets. Finally, principal component analysis was used to reduce the dimensionality of the data to two vectors, again by fitting only on the training set features and transforming all datasets.

### Machine learning analysis

The machine learning analysis was performed using the scikit-learn Python package (25). Different novelty detection algorithms were employed for the analysis, both independently and with a majority voting the ensemble approach: isolation forest (IF), local outlier factor (LOF), and one-class SVM (oSVM). These were fit on the inlier training dataset in an unsupervised fashion. Then, their performance was assessed separately on the inlier and outlier test sets. The predictions made on each test set case were then recorded and combined, together with the ground truths, to build confusion matrices and obtain accuracy metrics.

### Statistical analysis

All statistical tests were conducted in R (R for Unix/Linux, version 3.4.4, the R Foundation for Statistical Computing, 2014). Continuous data are presented as mean and standard deviation. Categorical and ordinal data are presented as value counts and proportions. The Shapiro–Wilk test was used to assess the normality of distribution of continuous data. Analysis of variance and Fisher exact tests were used to assess for differences in variable distribution among the training and test groups. Precision (i.e., positive predictive value), recall (i.e., sensitivity), accuracy (n correct predictions/all cases), and f-score (i.e., harmonic average of precision and recall) were calculated as accuracy metrics.

## Results

One hundred and fifty-five patients were enrolled in the study (M:F = 81:74 = 1.1; median age = 48.7 years; range = 18–78 years). Regarding the pituitary adenomas’ features, 129 (83%) were macroadenomas and 26 (17%) were microadenomas; 81 were nonfunctioning tumors (52%), while 43 (27%) were GH secreting, 15 (9.6%) were adrenocorticotropic hormone (ACTH) producing, 12 (0.6%) were prolactinomas, and finally 3 (0.2%) were GH/prolactin (PRL)-secreting adenomas. According to Micko grading scale, i.e. modified Knosp classification (26), we found that 22 (14.2%) were grade 0, 22 (14.2%) were grade 1, 44 (28.4%) were grade 2, 27 (17.4%) were grade 3A, and 10 (0.6%) were grade 4. Among functioning tumors, we noted that 36 (23.2%) had received prior medical treatment (Table 1).

**Table 1:** Patient characteristics.

		Training set	Inlier test set	Outlier test set	*p*-value
Age (years)		47.91 (±13.79)	47.69 (±15.92)	50.62 (±15.18)	0.6650
Sex	M	59	17	5	0.7256
	F	56	12	6	
Knosp	0	15	6	1	0.0015
	1	20	2	0	
	2	29	11	4	
	3	23	3	1	
	4	3	2	5	
Status	Nonfunctioning	55	17	9	0.0733
	Functioning	60	12	2	
Status PRL	0	102	29	11	0.1058
	1	13	0	0	
Status GH	0	82	19	11	0.0663
	1	33	10	9	
Status GH-PRL	0	112	29	11	1.0000
	1	3	0	0	
Size	micro	21	4	1	0.8063
	macro	94	25	10	
Preoperative treatment	0	84	24	11	0.0790
	1	31	5	0	
MRI barrier: strong	0	30	6	11	1.405 × 10^−6^
	1	85	23	0	
MRI barrier: weak	0	111	28	3	4.487 × 10^−8^
	1	4	1	8	
Intraoperative barrier: strong	0	29	4	11	2.788 × 10^−7^
	1	86	25	0	
Intraoperative barrier: weak	0	109	28	1	2.191 × 10^−10^
	1	6	1	10	

The distribution according to the sellar barrier subtype on MRI was as follows: 108 (69.7%) adenomas had a strong barrier, 13 a weak barrier (0.8%) (Figures 1–3), and finally 34 (28.1%) had a mixed barrier; as per the intraoperative findings, we observed that 111 (71.9%) had a strong barrier, 17 (12.5%) had weak barrier, and 27 (15.6%) had mixed barrier (Figures 1–3 and Table 1).

**Figure 4:**
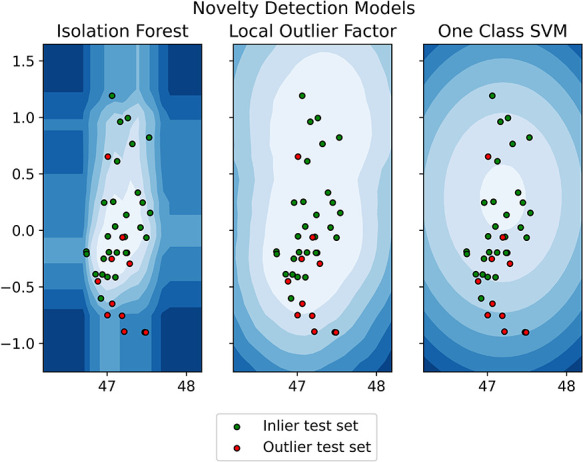
Model's decision function in relation to the distribution of inlier and outlier test set patients.

The training dataset included 115 (74%) patients without intraoperative fistula, while the inlier and outlier test sets included 29 (19%) and 11 (7%) patients, respectively. Patient clinical and demographic data are presented in Table 1. Significant differences between the three datasets were found for Knosp classification score (*p* = 0.0015), MRI barrier: strong (*p* = 1.405 × 10^−6^), MRI barrier: weak (*p* = 4.487 × 10^−8^), intraoperative barrier: strong (*p* = 2.788 × 10^−7^), and intraoperative barrier: Weak (*p* = 2.191 × 10^−10^). Accuracy, sensitivity, and specificity for outlier detection were 0.70, 0.64, and 0.72 for IF, 0.85, 0.45, and 1.00 for LOF; 0.83, 0.64, and 0.90 for oSVM; and 0.83, 0.55, and 0.93 for the ensemble. Confusion matrices and accuracy metrics are presented in Table 2. Figure 4 shows a plot of each model's decision function in relation to the distribution of inlier and outlier test set patients (Figure 4).

**Table 2:** Accuracy metrics.

Isolation forest				
Accuracy: 0.7				
			ML	
			1	0
Class	1		7	4
	0		8	21
	Precision	Recall	F1	Number
Outliers	0.47	0.64	0.54	11
Inliers	0.84	0.72	0.78	29
Macro average	0.65	0.68	0.66	40
Weighted average	0.74	0.70	0.71	40
Local outlier factor
Accuracy: 0.850
			ML	
			1	0
Class	1		5	6
	0		0	29
	Precision	Recall	F1	Number
Outliers	1.00	0.45	0.62	11
Inliers	0.83	1.00	0.91	29
Macro average	0.91	0.73	0.77	40
Weighted average	0.88	0.85	0.83	40
One-class SVM
Accuracy: 0.825
			ML	
			1	0
Class	1		7	4
	0		3	26
	Precision	Recall	F1	Number
Outliers	0.70	0.64	0.67	11
Inliers	0.87	0.90	0.88	29
Macro average	0.78	0.77	0.77	40
Weighted average	0.82	0.82	0.82	40
Ensemble
Accuracy: 0.825
			ML	
			1	0
Class	1		6	5
	0		2	27
	Precision	Recall	F1	Number
Outliers	0.75	0.55	0.63	11
Inliers	0.84	0.93	0.89	29
Macro average	0.80	0.74	0.76	40
Weighted average	0.82	0.82	0.82	40

## Discussion

The possibility to predict outcomes is critical to ensure the highest standards of surgical care, above all to satisfy patient inquiries with regard to the pros and cons of the procedure they are about to undergo.

CSF leak is one of the most threatening complications of transsphenoidal pituitary surgery, and also per its related potential complications, such as meningitis (27) or tension pneumocephalus (28).

Years of peculiar care and efforts, in terms of materials and reconstruction techniques, have been given to this issue (5, 6, 29–35), which nowadays has been reported as low as 2% among experienced groups (36–39).

It is crucial to highlight that albeit intraoperative CSF leak rates are reported as high as 10.3%–69%, postoperative CSF leak rates lower down to 1.3%–8% (38, 40–54).

In the present case series, we found a rate of intraoperative CSF leak of 7.1% and postoperative CSF leak of 0%, which are similar to those reported in the literature for this kind of surgery. These findings suggest that the rate of patients with intraoperative CSF leak who finally developed a postoperative CSF leak is negligible; this is mostly because of the improvement of reconstruction techniques over the years and the refinement of surgical skills in skull base surgeons (12).

However, there are only few reports concerning the possible risk factors that can be detected preoperatively to predict an intraoperative CSF leakage, but univocal consensus has not achieved yet: different groups claimed an increased risk of postoperative fistula upon the opening of the third ventricle, or in cases of patients with higher BMI (8–11).

The *sellar barrier concept* and its role in predicting the risk of intraoperative CSF leakage was recently introduced (12, 13) and confirmed in a clinical multicentric study (14).

### Predictive factors of intraoperative CSF leak

In the present study, none of the patients presented intraventricular invasion, CSF leak occurred indifferently in patients with high or low/normal BMI, and all procedures were performed by expert neurosurgeons familiar with pituitary surgery, *via* a standard corridor.

The authors consider crucial the ability of predicting an intraoperative CSF leak though the sellar barrier concept is effective. The sellar barrier concept fills an empty space for contemporary literature on this topic.

We provided an analysis of the risk of postoperative CSF leak in three different classes of patients undergoing endoscopic endonasal removal of pituitary adenomas, by means of an ML model; classes were defined according to the MRI appearance of the so-called “sellar barrier.”

### Sellar barrier as predictor of CSF leak in endoscopic pituitary surgery

In our initial publication, we demonstrated the correlation between the intraoperative classification of the sellar barrier and the presence of intraoperative CSF leak (12). Later, in a second publication, we found a correlation between the MRI classification of the sellar barrier and the presence of CSF leak (13). In a recent multicentric study (14), this relation has been confirmed: albeit without ML analysis, patients assessed as at higher risk of intraoperative CSF leakage would benefit most from a gentle dissection of the most superior aspects of the tumor, paying attention to preserve as much as possible the layer of the gland to cover the diaphragm.

In the present study, we found that the preoperative MRI classification could predict the risk of intraoperative CSF fistula, as confirmed also by ML analysis.

Our ML model identified with outstanding accuracy that there is a cogent correlation between the weak barrier type and the intraoperative CSF leakage MRI barrier: strong (*p* = 1.405 × 10^−6^), MRI barrier: weak (*p* = 4.487 × 10^−8^), intraoperative barrier: strong (*p* = 2.788 × 10^−7^), and intraoperative barrier: weak (*p* = 2.191 × 10^−10^).

These findings are relatively new and might provide further issue to be considered when defining the surgical planning. Hence, also per intraoperative observation, a weak barrier represents a “*locus minoris resistentiae*,” whose careless manipulation during tumor removal can expose the increased risk of intraoperative CSF leakage.

In the near future, this computer-aided decision-making tool might improve surgical quality by regularly identifying those patients at higher risk of developing intraoperative CSF leakage and related complications; validated machine learning tools might change routine surgical practice if properly setup: the creation of a computerized predictive algorithm can be a crucial step to further refine modern neurosurgery.

### Clinical–surgical application

Our teams are working on a risk classification of intraoperative CSF leak to refine the most appropriate surgical strategy and adequately inform the patient about its postoperative course.

Claude Bernard used to say, “who doesn't know what he's looking for, doesn't understand what he finds.” The clinical application of the sellar barrier concept will allow the skull base surgery team to predict the scenario they will encounter. Thanks to this, you will be able to carefully select the method to be used in the reconstructive phase.

During preoperative consultation, the use of imaging software allows the surgeon to show the sellar barrier to the patient and explain with a graphic support about their risk of CSF leakage. Thanks to this, the surgeon can speak clearly and precisely with the patient. It allows the surgeon to inform the patient about his/her possible postoperative evolution: postoperative nasal symptoms, surgery time, and the risk of postoperative CSF fistulae, among others. This type of information would have legal implications in the postoperative period (12–14).

### Limitations

The concept of the sellar barrier cannot be considered as a totally independent predictor factor of CSF leakage.

This is a prospective cohort study with a small series of patients. A multicenter study with more extensive patient series is required to validate this concept and its clinical applicability.

Finally, it is worth reminding that the downside of the ML model can be its troublesome application to a clinical context: the interpretation of this model conceives the human–machine interaction as its highest moment.

## Conclusions

The sellar barrier is a new parameter to be considered in the risk assessment of intraoperative CSF leak. The present study demonstrates the efficacy of the sellar barrier concept in patients operated for endoscopic endonasal pituitary removal and strengthens its clinical applicability.

There is a true correlation between the type of *sellar barrier* at MRI and its *in vivo* features as observed during endoscopic endonasal surgery. The novelty detection models highlighted differences between patients who developed an intraoperative CSF leak and those who did not.

## Data Availability

The original contributions presented in the study are included in the article/Supplementary Material, further inquiries can be directed to the corresponding author.
